# Acute mesenteric ischemia in COVID-19: Case report and current understanding

**DOI:** 10.5339/qmj.2022.11

**Published:** 2022-02-28

**Authors:** Saurabh Chandrakar, Priyanka Sangadala, Megha Gupta, Deepak Kumar A, Ankit Agarwal

**Affiliations:** Department of Anaesthesia and Critical Care. AIIMS Rishikesh, Uttarakhand, India. E-mail: docmeghagupta@gmail.com

**Keywords:** COVID-19, SARS-COV-2, Mesenteric ischemia, Thrombosis

## Abstract

Introduction: Since the outbreak of coronavirus disease 2019 (COVID-19) in December 2019, various thrombotic complications have been frequently reported in patients with infection. Acute mesenteric ischemia (AMI) is a rare but life-threatening complication in this disease, which requires early recognition and prompt treatment.

Case presentation: We report two cases of COVID-19-related AMI. Both patients underwent emergency laparotomy for small bowel ischemia. The first patient received prompt intervention and was discharged 5 days after surgery. The second patient presented late to the hospital and succumbed 72 h after surgery.

Conclusion: These two cases highlight the importance of high suspicion, early recognition, and prompt treatment in patients with abdominal symptoms related to COVID-19.

## Introduction

Severe acute respiratory syndrome coronavirus 2 (SARS-CoV-2) emerged in Wuhan City, China, in December 2019. The novel coronavirus disease2019(COVID-19) became a global pandemic over a few months and destabilized societies worldwide.^1^

Primarily, this disease is known for its pulmonary involvement; a subset of patients demonstrates clinically remarkable hypercoagulability.^2^ Thrombotic manifestations range from venous thrombosis, acute pulmonary embolism to arterial thrombosis involving cerebral, coronaries, extremity, and mesenteric vessels.^3^ Acute mesenteric ischemia (AMI) is a potentially fatal vascular emergency with an overall mortality of 60%–80%.^4^ Patients with severe COVID-19 with hemodynamic instability are at increased risk of developing AMI.^5^ Herein, we report two cases of mesenteric ischemia in patients with COVID-19.

The institutional ethics committee approved this case series (IEC/21/304), and informed consent was obtained from the patients’ relatives. This manuscript reinforces the importance of keeping a very low threshold for suspicion of COVID-19-related AMI in patients presenting with gastrointestinal symptoms. Moreover, it emphasizes early referral to a higher center and early intervention to improve the outcomes.

## Presentation Of Cases

### Case 1

**Figure 1.**
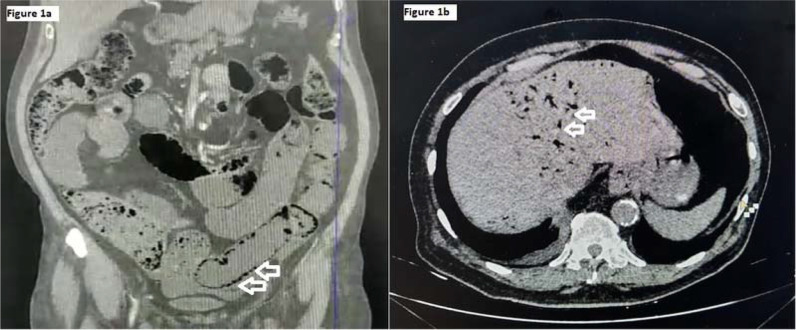
Computed tomography angiography of the abdomen showing pneumatosis intestinalis (Fig. 1A) and air in the portal vein (Fig. 1B) (marked with white arrows)

A 72-year-old man with known comorbidities of hypertension, diabetes, and chronic kidney disease (stage III) was transferred from the ward to the critical care unit for severe abdominal pain that started 12 hearlier and worsened in the next 6 h. The patient also had three episodes of non-projectile biliary vomiting, non-passage of stools, and worsening breathlessness. Ten days before this episode, the patient tested positive for SARS-CoV-2 by real-time polymerase chain reaction (RT-PCR). On physical examination, the patient was afebrile, had increased respiratory rate (30 breaths per minute) with oxygen saturation of 91%, and received 6 L/minute of oxygen via face mask. The heart rate was 90 beats per minute (bpm), and the blood pressure was 100/60 mm Hg. He had diffused abdominal pain, which aggravated with deep palpation in all abdominal regions. Laboratory investigations revealed neutrophilic leukocytosis (21000/μL white blood cells, neutrophils 92%), elevated D-dimer (2.2 mg/L), hypercholesterolemia (204 mg/dL), and elevated serum lactate level(7mmol/L). Liver function test and amylase levels were within the normal range. On chest X-ray imaging, bilateral ground-glass opacities with right basal consolidation were present with no air below the diaphragm. On computed tomography angiography (CTA) abdomen, pneumatosis intestinalis (Figure 1A) and air in the portal vein (Figure 1B) were visualized. Immediately, the patient was taken for emergency laparotomy, and gangrenous bowel was resected, and end-to-end anastomosis was completed. The patient was on total parenteral nutrition for 2days. With the return of bowel movements, enteral nutrition was started. After 5 days, the patient was discharged with aspirin 75 tablet mg once daily for 6 weeks.

### Case 2

**Figure 2.**
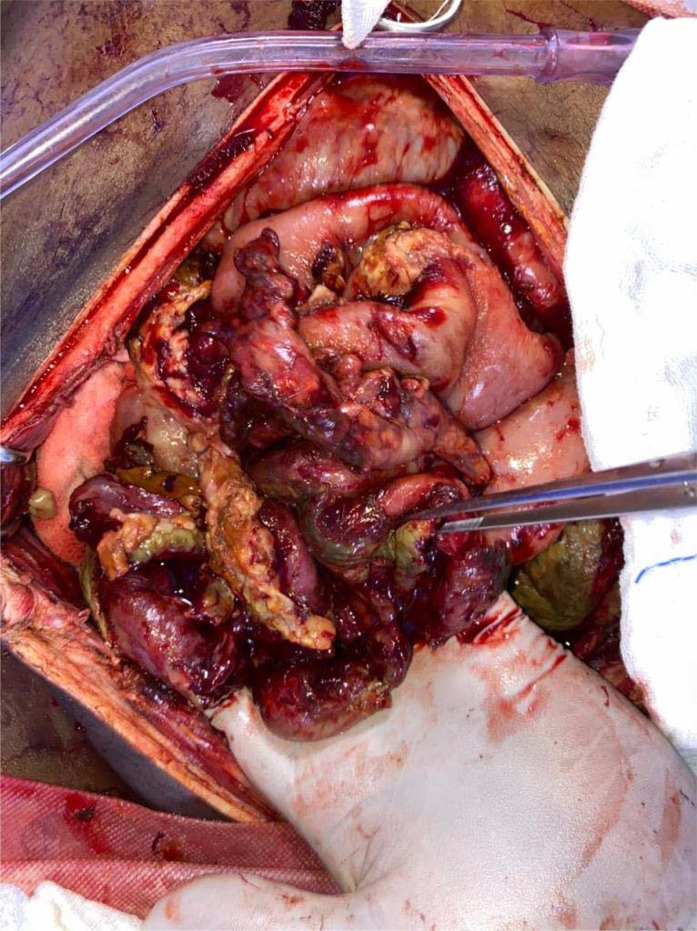
Ischemic, gangrenous bowel was located 25 cm proximal to the ileocecal valve, which was resected, and ileostomy was performed.

A 70-year-old woman with known comorbidities of uncontrolled hypertension and diabetes (on irregular medicines) presented to the emergency department with diffuse abdominal pain, difficulty in passing stool and flatus, and projectile bilious vomiting from the past 96 h. She was initially managed conservatively at a local hospital for the above symptoms. However, with the deterioration of her clinical status, she was transferred to our hospital. Three weeks before the onset of illness, the patient tested positive for SARS-CoV-2 by RT-PCR and currently was in the recovery phase. On examination, the patient was in shock (blood pressure of 85/50 mm Hg, pulse rate of 130 bpm, prolonged capillary refilling time) and respiratory distress (respiratory rate of 30 breaths per minute with the use of accessory muscles), for which noninvasive ventilator support, fluid resuscitation, and vasopressors were initiated. Laboratory investigations revealed neutrophilic leukocytosis (white blood cells, 23000/μL; neutrophils, 96%), elevated D-dimer (13 mg/L), elevated lipase (300U/L), elevated amylase (240 U/L), and elevated serum lactate level (9 mmol/L). Arterial blood gas showed high anion gap metabolic acidosis. On chest X-ray imaging, bilateral ground-glass opacities with right basal consolidation were present with no air below the diaphragm. Bedside ultrasonography showed dilated bowel loops with free fluid in the pelvis. Because of progressive worsening of the clinical situation, the patient was taken for emergency laparotomy. Intraoperatively, ischemic, gangrenous bowel was located 25 cm proximal to the ileocecal valve, which was resected, and ileostomy was performed (Figure 2). The patient was moved to the critical care unit to manage shock, metabolic acidosis, and ventilator support. The patient developed worsening shock, metabolic acidosis, and acute kidney injury, and on postoperative day 3, she succumbed to her illness because of multiple organ dysfunction syndrome.

## Discussion

In COVID-19, excessive inflammation, hypoxia, immobilization, sepsis, liver injury secondary to angiotensin-converting enzyme 2 (ACE2) receptor expression, and diffuse intravascular coagulation can predispose patients with critical illness to arterial and venous thromboembolism.^6^ AMIis a rare abdominal emergency, with incidence of 0.09%–0.2% in the general population and 1.9%–3.8% in patients with COVID-19, and is associated with high morbidity and mortality rates.^7,8^

The putative mechanisms, in isolation or in varying combinations, that can account for AMI in COVID-19 include (a) a coagulation disorder (hypercoagulability) induced by an inflammatory state, endothelial activation, hypoxia, and immobilization; (b) elevated levels of von Willebrand factor released from Weibel–Palade bodies in response to endothelial damage; (c)intestinal tropism and direct bowel damage because of ACE2 expression on enterocytes of the small bowel; and (d) shock or hypoperfusion leading to non-occlusive mesenteric ischemia.^9^

The clinical symptoms of patients with COVID-19-relatedAMI include nausea, vomiting, abdominal pain, distension, constipation, loose stool, breathlessness, and fever.^10^

Laboratory findings are nonspecific and may be elevated in COVID-19 without ongoing mesenteric ischemia. There is wide heterogeneity in the studies reporting various acute phase reactants. In a meta-analysis involving 75 patients with COVID-19-related mesenteric ischemia, D-dimer was the most commonly elevated serum acute phase reactant (97.1%), followed by C-reactive protein (79.2%) and serum leukocyte count (70.8%). Death and discharge were recorded in equal frequency (47%) when those with elevated D-dimer levels were described. In patients with high C-reactive protein, death was recorded in 57.9% and discharge in 42.1%of the cases.^11^ Although blood tests may reveal elevated levels of these reactants in AMI, they are nonspecific and may be elevated in severe COVID-19 infection even without AMI.^6^ Radiography and ultrasonography are nonspecific. CTA is the gold standard to diagnose AMI. Considering that chest CT scans are frequently performed in patients with COVID-19, it might be reasonable to extend them to the abdomen, specifically in patients who present with abdominal pain or have an unfavorable disease course.^12^ The presence of gas in the portal vein and intramural bowel gas (also known as pneumatosis intestinalis) in CTA of the abdomen are specific for intestinal ischemia. Pneumatosis intestinalis can also be caused by obstructive pulmonary disease or asthma, systemic autoimmune diseases, inflammatory bowel diseases, organ transplantation, barotrauma (during ventilation) and other iatrogenic causes (post-endoscopy/colonoscopy, post-surgery), and use of some medications, including corticosteroids and chemotherapeutic agents.^13^ On the pooled analysis conducted by Ojha et al., small bowel ischemia (46.67%) was the most prevalent abdominal CT finding among patients diagnosed with mesenteric ischemia in COVID-19. However, ischemic colitis was a more prevalent diagnosis in the two case series reported by Bhayana et al.^3^ and Norsa et al.^14^

The goal of treatment for patients with AMI is to restore intestinal blood flow as rapidly as possible after initial management that includes systemic anticoagulation and empiric broad-spectrum antibiotic therapy, among others. Specific treatment depends on the clinical status of the patient and the etiology and location of the occlusion.^15^ Initial catheter-based arteriography and possible endovascular treatment (mechanical thrombectomy or balloon angioplasty) can only be considered for patients with stable hemodynamics with no clinical signs of advanced ischemia.^16^ Patients with AMI manifesting clinical symptoms or signs of progressive ischemia (e.g., peritonitis, sepsis, and pneumatosis intestinalis) warrant immediate surgery.^17^

Unfortunately, many patients with advanced ischemia who underwent surgery with SARS-CoV-2 infection had a higher risk of postoperative death than patients who were not infected.^18^ To decrease the morbidity and mortality of AMI in COVID-19, physicians should have a high index of suspicion so that timely intervention can be performed. Moreover, information about the warning signs of acute problems of COVID-19 should be provided at the community level to prevent delayed referral of the patients, who should implement the necessary recommendations.^19^

## Conclusion

The first patient had a good postoperative course because of early recognition and treatment. On the contrary, the second patient had a poor outcome because of delayed presentation. Mesenteric ischemia should be highly suspected in patients with COVID-19 presenting with gastrointestinal symptoms (severe progressive abdominal pain, nausea, vomiting, fever, constipation, loose stool, breathlessness, or weight loss) to perform timely diagnostic testing and appropriate decision-making. Since chest CT is frequently performed in patients with COVID-19, it might be reasonable to extend them to the abdomen, specifically in patients with abdominal pain or an unfavorable disease course. Information about the warning signs of acute problems of COVID-19 should be provided at the community level to prevent delayed referral of the patients.

